# Tannic acid reduced apparent protein digestibility and induced oxidative stress and inflammatory response without altering growth performance and ruminal microbiota diversity of Xiangdong black goats

**DOI:** 10.3389/fvets.2022.1004841

**Published:** 2022-09-08

**Authors:** Zuo Wang, Lei Yin, Lei Liu, Xinyi Lan, Jianhua He, Fachun Wan, Weijun Shen, Shaoxun Tang, Zhiliang Tan, Yanming Yang

**Affiliations:** ^1^College of Animal Science and Technology, Hunan Agricultural University, Changsha, China; ^2^CAS Key Laboratory of Agro-Ecological Processes in Subtropical Region, National Engineering Laboratory for Pollution Control and Waste Utilization in Livestock and Poultry Production, Hunan Provincial Key Laboratory of Animal Nutrition & Physiology and Metabolism, Institute of Subtropical Agriculture, Chinese Academy of Sciences, Changsha, China; ^3^Jiurui Biology & Chemistry Co., Ltd., Zhangjiajie, China

**Keywords:** tannic acid, goats, oxidative stress, inflammatory response, ruminal microflora

## Abstract

The present study was performed to evaluate the impacts of tannic acid (TA) supplementation at different levels on the growth performance, physiological, oxidative and immunological metrics, and ruminal microflora of Xiangdong black goats. Twenty-four goats were randomly assigned to four dietary treatments: the control (CON, basal diet), the low-dose TA group [TAL, 0.3 % of dry matter (DM)], the mid-dose TA group (TAM, 0.6 % of DM), and the high-dose TA group (TAH, 0.9 % of DM). Results showed that the growth performance was unaffected (*P* > 0.05) by adding TA, whilst the 0.3 % and 0.6 % TA supplementation significantly decreased (*P* < 0.05) the apparent digestibility of crude protein (CP) and ruminal NH_3_-N concentration, and raised (*P* < 0.05) the level of total volatile fatty acid (TVFA) in rumen. The increments of alanine aminotransferase (ALT), triglyceride (TG), cortisol (CORT), total antioxidant capacity (T-AOC), interleukin (IL)-1β, IL-6, and serumamyloid A (SAA), and decrements of globulin (GLB), immunoglobulin G (IgG), cholinesterase (CHE), glutathione reductase (GR), creatinine (CRE), growth hormone (GH), high-density lipoprotein cholesterol (HDLC), and insulin-like growth factor 1 (IGF-1) to different extents by TA addition were observed. Although the Alpha and Beta diversity of rumen bacterial community remained unchanged by supplementing TA, the relative abundance of the predominant genus *Prevotella_1* was significantly enriched (*P* < 0.05) in TAL. It could hence be concluded that the TA supplementation in the present trial generally decreased CP digestion and caused oxidative stress and inflammatory response without influencing growth performance and ruminal microbiota diversity. More research is needed to explore the premium dosage and mechanisms of effects for TA addition in the diet of goats.

## Introduction

Tannins are water-soluble bioactive polyphenols capable of forming complexes basically with proteins and carbohydrates *via* hydrogen bonds (1, 2). Precedent investigations have shown that tannins could improve nitrogen digestion and reduce methane yield of ruminants (1, 3–5). Tannins can bind to the feed protein in the rumen and thence protect protein against ruminal microbial degradation, leading to the increase of dietary protein and amino acids entering the small intestine (2, 6). Moreover, the reduction in methane emission by tannins could be achieved through decreasing fiber digestion and interfering with rumen microorganisms (1, 5, 7).

Tannins, depending on the structure and reactivity, are mainly categorized into condensed tannins (CTs) and hydrolysable tannins (HTs) (8). It is noteworthy that the bioactive influences of tannins on ruminants might vary due to different sources, types, doses, animals, and rations (4, 9–11). Although the quantity of rumen undegradable protein (RUP) would be enhanced by tannins, the net flux of metabolizable protein (MP) into the small intestine could not definitely be raised, as tannins might also suppress the microbial protein synthesis within rumen (12). Mezzomo et al. (13) found that adding CT from quebracho extract (0.4 % of DM) increased the flow of RUP and MP, and crude protein digestibility in beef steers fed a high-concentrate (87% of DM) diet with soybean meal as the true protein source. Oppositely, the decline in feed crude protein digestion of dairy cattle was also reported, when 0.15 and 0.4 % quebracho CT were respectively supplemented in a 34% concentrate ration (14).

In contrast to the CTs (1,900–28,000 Da), HTs have lower molecular weight (500–3,000 Da) and less binding capacity, which makes HTs more readily decomposed and absorbed in the digestive tract and potentially exert toxic effects on ruminants (8). Nonetheless, no harmful influences of chestnut-originated HT was observed either in sheep with the supplemental levels at 0.15 and 0.3% (3), or in beef cattle at 0.25 and 1.5% of DM (8). As a typical HT, tannic acid (TA) consists of 8–10 molecules of gallic acid per molecule of glucose, making its structure simpler than those of the CTs (15). The drop in methane production of bulls by adding 0.65, 1.3, and 2.6% TA was previously reported (5). Subsequently, Zhou et al. (15) found that the emission of urea and N_2_O-N in the urine of steers decreased in response of the supplementation of 1.69% TA. Furthermore, the antioxidative property of TA has been verified in previous studies involving antioxidant assays (16) or *in vivo* trials in rats (17, 18), implying that TA could probably act as a promising antioxidant additive for ruminants.

So far, methane production and nitrogen utilization have constantly remained the two primary topics in research targeting the influences of TA on ruminants, and the majority of studies on supplementing TA in the ration for ruminants have been conducted on cattle and sheep (2, 5, 6, 15, 19). By contrast, information referring to the responses of goats in digestion, performance, and physiological function to TA supplementation is rare. Moreover, despite the fact that the influences of tannins on the ruminal microbes have been explored precedently (20, 21), it is still necessary to deeply reveal the effects of TA at different doses on the rumen microbiome using the full-length 16S rRNA gene sequencing with better precision than the partial 16S rRNA gene sequencing, in view of the essential role of rumen microflora in the performance and health of ruminants (22, 23).

Therefore, the objective of the present study was to assess the impacts of TA supplementation at different levels on the growth performance, physiological, oxidative and immunological metrics, and ruminal microbiota of black goats, and hence offer more detailed references for the application of TA in the ruminant industry.

## Materials and methods

### Animals, diets, and management

All procedures involving animals in the present experiment was approved by the Animal Care Committee (approval number: 20210906), College of Animal Science and Technology, Hunan Agricultural University, Changsha, China. Twenty-four male Xiangdong black goats (*Capra hircus*) averaging 12 ± 1.6 kg of body weight, and 8 ± 1 months of age were randomly allocated to each of the four dietary treatments including the control group (the basal TMR ration, CON), the low-dose TA group (the basal ration supplemented with 0.3 % TA, TAL), the mid-dose TA group (the basal ration supplemented with 0.6 % TA, TAM), and the high-dose TA group (the basal ration supplemented with 0.9 % TA, TAH), in a single-factor design. Six goats were included in each treatment group. The components and nutritional compositions of the basal TMR diet are displayed in Table 1. The TA (purity 97 %) used in this study was a commercial product isolated from the *Rhus chinensis* Mill. (Jiurui Biology & Chemistry Co. Ltd., Zhangjiajie, China). All the goats were housed individually and fed *ad libitum* twice per day (08:00 h and 16:00 h) with free access to fresh water. This trial lasted for 97 days, consisting of 7 days of adaptation and 90 days of data and sample collection.

**Table 1:** Components and nutritional composition of the basal TMR ration.

**Ingredients** ^a^ **, g/kg DM**		**Nutritional composition** ^b^ **, g/kg DM**	
Rice straw	310	NE_m_, Mcal/kg DM	1.33
Alfalfa hay	190	OM	891.3
Corn meal	340	CP	73.9
Soybean meal	95	EE	18.6
Wheat bran	20	NDF	310.9
Premix	45	ADF	150.8
		Ca	12.3
		P	2.9

a Every 1 kg of premix contained 2,800 mg of Zn, 2,200 mg of Mn, 1,500 mg of Fe, 980 mg of Cu, 36 mg of I, 16 mg of Co, 15 mg of Se, 2,400 mg of vitamin B3, 520000 IU of vitamin A, 160000 IU of vitamin D3, 1600 IU of vitamin E.

b NEm, net energy for maintenance.

### Sampling

Throughout this trial, the TMR feed and the leftover for each goat were sampled every 5 days. The total feces from each goat was collected from 91 d to 97 d. The rumen liquid from the central rumen was sampled through the oral cavity 4 h after morning feeding on 97 d, using the previously described methods (24, 25). The serum and plasma samples were obtained as reported by Wang et al. (26), 4 h after morning feeding on 97 d. After collection, all the samples were instantly frozen in liquid nitrogen and stored at −80°C until subsequent analysis.

### Chemical and biochemical analysis

The contents of dry matter (DM; method 930.15), ash (method 942.05), crude protein (CP; method 2001.11), ether extract (EE, method 920.39), neutral detergent fiber (NDF; method 2002.04), and acid detergent fiber (ADF; method 973.18) in the feed and feces were measured by following the instructions of AOAC (2005). The calcium (Ca) and phosphorus (P) in the basal ration were analyzed as introduced previously (26, 27). The assessment for pH, ammonia nitrogen (NH_3_-N), and volatile fatty acid (VFA) of the rumen fluid was conducted as reported in prior studies (23, 28).

For the measurement of lipopolysaccharide (LPS) endotoxin in the rumen fluid and plasma, and relevant inflammatory indicators, methods illustrated by Wang et al. (26, 29) were adopted. A Roche Cobas automatic biochemistry analyzer (c311, Roche Ltd., Basel, Switzerland) and related specific kits (Roche Ltd., Basel, Switzerland) were employed to determine the biochemical blood characteristics, by referring to the manufacturer's protocols and precedent report (30).

### DNA extraction, PCR amplification, and full-length 16S rRNA gene sequencing

The genomic DNA from the rumen fluid samples was isolated through a phenol-free bead-beating approach depicted by Yu and Morrison (31). Afterwards, the PCR amplification for the full-length bacterial 16S rRNA genes was performed using the universal primers 27F (5'-AGRGTTTGATYNTGGCTCAG-3') and 1492R (5'-TASGGHTACCTTGTTASGACTT-3') with barcode, with the detailed procedures set as introduced previously (23). The amplicon sequencing library was prepared and examined using the methods in precedent reports (23, 25). Finally, the amplicon library was sequenced on the PacBio Sequel II platform (Pacific Biosciences, Menlo Park, USA) and single-end reads were generated.

### Bioinformatics analysis

The bioinformatics analysis in this study was conducted with the assistance of the BMK Cloud (Biomarker Technologies Co., Ltd., Beijing, China). The pretreatment for the raw sequencing data, including the circular consensus sequencing (CCS) reads recognition, CCS reads quality filtering, and chimera sequence removal, were successively performed following processes in our previous investigations (23, 25, 32, 33). Subsequently, the operational taxonomic unit (OTU) clustering, OTU taxonomy assignment, and OTU abundance normalization were carried out in sequence as introduced previously (23, 25, 32, 33). The QIIME (V1.9.1) and R software (V3.1) were used to fulfill the analysis of the Alpha diversity and Beta diversity, and the function prediction of Tax4Fun was achieved by using approaches in prior reports (23, 25, 32–34). All the raw sequences acquired in the present experiment were deposited to the sequence read archive (SRA) of the NCBI database with the accession number PRJNA847688.

### Statistical analysis

In order to evaluate the impacts of supplementing TA at different levels on the growth performance, nutrient digestibility, rumen fermentation characteristics, physiological and immunological parameters, and Alpha diversity indices, the relevant data were analyzed through the single-factor ANOVA statistical analysis, followed by the Duncan's multiple range test using the SPSS statistics (V23.0, IBM Corporation, Armonk, USA). The orthogonal polynomial contrasts were used to analyze the linear and quadratic effects of TA dose. Least squares means are reported throughout the text. Statistical difference was respectively declared as significant or highly significant at *P* < 0.05 or *P* < 0.01, while trend was discussed at 0.05 < *P* ≤ 0.10. Linear discriminant analysis effect size (LEfSe) was adopted to compare relative abundances of microbial taxa across treatments, and significant differences were considered by a linear discriminant analysis (LDA) score > 4.0 and *P* < 0.05.

## Results

### Growth performance and apparent nutrient digestibility

None of the DM intake (DMI), average daily gain (ADG), and feed conversion ratio (FCR) of black goats was affected by the inclusion of TA (*P* > 0.05) (Table 2). As for the apparent nutrient digestibility, supplementing TA reduced the apparent digestibility of CP in a quadratic manner (*P* < 0.05), with the minimum of CP digestibility present in the TAH group. In contrast, no significant effect of TA addition on the apparent digestibilities of the remaining nutrients was observed (*P* > 0.05).

**Table 2:** Effects of tannic acid at different doses on growth performance and apparent nutrient digestibility of black goats.

**Items^1^**	**Treatments** ^2^				**SEM^3^**	***P*** **-value** ^4^		
	**CON**	**TAL**	**TAM**	**TAH**		**Dose**	**L**	**Q**
Growth performance								
DMI, kg/d	0.74	0.71	0.76	0.69	0.124	0.331	0.463	0.627
ADG, kg/d	0.12	0.12	0.13	0.12	0.021	0.360	0.703	0.847
FCR	5.99	6.24	6.04	5.92	1.087	0.837	0.722	0.726
Apparent nutrient digestibility, %								
DM	66.36	67.83	63.97	64.34	2.834	0.287	0.168	0.383
OM	70.86	73.17	68.52	69.64	2.598	0.083	0.199	0.420
CP	70.82^a^	67.19^a^^b^	55.33^b^^c^	52.00^c^	8.006	0.044	0.004	0.020
EE	67.68	72.84	63.93	70.49	5.773	0.756	0.986	0.993
NDF	57.36	53.86	54.99	49.53	4.033	0.364	0.104	0.270
ADF	48.91	44.90	49.05	35.56	7.472	0.054	0.055	0.079

1 FCR, feed conversion ratio.

2 CON, control group; TAL, low-dose tannic acid treatment; TAM, mid-dose tannic acid treatment; TAH, high-dose tannic acid treatment.

3 SEM, standard error of means for treatments.

4 L, linear effect of the tannic acid dose; Q, quadratic effect of the tannic acid dose.

a, b, c Means within a row for treatments that do not have a common superscript differ (*P* < 0.05).

### Rumen fermentation parameters

The TA supplementation quadratically (*P* < 0.05) decreased the NH_3_-N concentration in the rumen fluid of black goats, and NH_3_-N amounts in both TAM and TAH were significantly lower than the CON (*P* < 0.05) (Table 3). The concentration of total volatile fatty acid (TVFA) rose quadratically (*P* < 0.05) as the TA level grew, peaking in both TAM and TAH compared to CON (*P* < 0.05). Besides, a quadratic (*P* < 0.05) increase in the proportion of valerate in response to TA addition was also noticed.

**Table 3:** Effects of tannic acid at different doses on rumen fermentation characteristics of black goats.

**Items^1^**	**Treatments** ^2^				**SEM^3^**	***P*** **-value** ^4^		
	**CON**	**TAL**	**TAM**	**TAH**		**Dose**	**L**	**Q**
pH	6.82	6.89	6.79	6.84	0.121	0.597	0.983	0.965
NH_3_-N	8.33^a^	8.00^a^^b^	7.87^b^	7.66^b^	0.392	0.021	0.002	0.008
TVFA	28.48^b^	29.02^b^	69.07^a^	68.17^a^	21.929	<0.001	<0.001	<0.001
VFA profile (mol/100 mol)								
Acetate	63.55	57.54	60.71	62.11	4.465	0.120	0.894	0.136
Propionate	19.22	19.56	18.15	19.71	2.249	0.670	0.987	0.824
Butyrate	9.87	12.62	11.07	10.35	2.379	0.225	0.982	0.222
Isobutyrate	2.87	3.81	3.51	2.93	0.807	0.131	0.941	0.071
Valerate	0.85^b^	1.50^a^^b^	1.82^a^	1.34^a^^b^	0.595	0.034	0.108	0.013
Isovalerate	3.65	4.97	4.74	3.57	1.271	0.124	0.841	0.054
A:P	3.42	2.96	3.35	3.20	0.519	0.462	0.779	0.760

1 TVFA, total volatile fatty acid; A:P, the ratio of acetate to propionate.

2 CON, control group; TAL, low-dose tannic acid treatment; TAM, mid-dose tannic acid treatment; TAH, high-dose tannic acid treatment.

3 SEM, standard error of means for treatments.

4 L, linear effect of the tannic acid dose; Q, quadratic effect of the tannic acid dose.

a, b Means within a row for treatments that do not have a common superscript differ (*P* < 0.05).

### Biochemical and physiological parameters of blood serum

Amongst all the blood biochemical and physiological parameters measured in this study, the globulin (GLB), immunoglobulin G (IgG), cholinesterase (CHE), glutathione reductase (GR), alanine aminotransferase (ALT), creatinine (CRE), triglyceride (TG), high-density lipoprotein cholesterol (HDLC), growth hormone (GH), cortisol (CORT), insulin-like growth factor 1 (IGF-1), and total antioxidant capacity (T-AOC) were influenced by the TA addition (*P* < 0.05) (Table 4). More specifically, supplementing goats with TA raised the levels of ALT (quadratic, *P* < 0.05), TG (quadratic, *P* < 0.05), CORT (quadratic, *P* < 0.1), and T-AOC (linear, *P* < 0.05), while decreased the densities of GLB (quadratic, *P* < 0.1), IgG (quadratic, *P* < 0.05), CHE (quadratic, *P* < 0.01), GR (quadratic, *P* < 0.01), CRE (quadratic, *P* < 0.01), HDLC (quadratic, *P* < 0.01), GH (quadratic, *P* < 0.05), and IGF-1 (quadratic, *P* < 0.1) to different extents.

**Table 4:** Effects of tannic acid at different doses on biochemical and physiological parameters in the blood serum of black goats.

**Items^1^**	**Treatments** ^2^				**SEM^3^**	***P*** **-value** ^4^		
	**CON**	**TAL**	**TAM**	**TAH**		**Dose**	**L**	**Q**
TP, g/L	66.3	71.7	72.2	70.5	6.86	0.477	0.319	0.285
ALB, g/L	36.7	34.8	36.1	37.6	3.33	0.563	0.539	0.402
GLB, g/L	16.5^a^	13.1^b^	15.0^a^^b^	13.7^b^	1.91	0.006	0.061	0.070
IgA, g/L	0.12	0.13	0.11	0.12	0.047	0.875	0.759	0.948
IgG, g/L	5.72^a^	3.44^d^	4.87^b^	4.13^c^	0.948	<0.001	0.056	0.018
CHE, U/L	98.5^a^	101.2^a^	100.4^a^	56.5^b^	28.93	0.010	0.015	0.004
GR, U/L	15.5^a^	14.7^a^	13.7^a^	8.0^b^	3.41	<0.001	<0.001	<0.001
ALT, U/L	3.74^b^	4.12^b^	6.67^a^	4.60^b^	1.562	0.002	0.077	0.028
AST, U/L	10.8	13.6	13.0	13.1	2.65	0.292	0.191	0.211
CRE, μmol/L	95.2^a^	98.4^a^	76.4^b^	71.0^b^	17.21	0.005	0.001	0.005
BUN, mmol/L	8.10	8.19	8.00	7.05	1.261	0.407	0.159	0.230
GLU, mmol/L	7.10	7.29	7.13	8.85	2.303	0.540	0.245	0.378
TG, mmol/L	0.11^b^	0.17^a^	0.21^a^	0.20^a^	0.055	0.002	0.001	0.001
TC, mmol/L	2.77	2.98	3.13	3.03	0.359	0.410	0.173	0.238
HDLC, mmol/L	0.92^a^	0.68^b^	0.60^b^	0.78^a^^b^	0.185	0.009	0.132	0.003
LDLC, mmol/L	1.46	1.39	1.67	1.48	0.272	0.342	0.512	0.722
HL, ng/mL	95.7	92.4	104.0	101.8	23.23	0.839	0.501	0.801
GH, ng/mL	4.79^a^	2.99^b^	3.85^a^^b^	4.41^a^	0.987	0.005	0.875	0.010
T3, ng/mL	6.77	5.63	6.49	5.70	1.341	0.397	0.358	0.634
T4, ng/mL	171.5	161.3	200.6	173.8	32.48	0.205	0.457	0.638
CORT, μg/mL	30.31^b^	39.82^a^	31.31^b^	31.91^b^	5.083	0.001	0.704	0.097
EPI, ng/mL	1.59	1.96	1.63	1.59	0.302	0.110	0.595	0.254
INS, mU/L	28.5	28.5	29.3	26.6	6.84	0.935	0.712	0.843
GC, ng/mL	0.23	0.29	0.26	0.25	0.048	0.262	0.817	0.288
IGF-1, ng/mL	248.3^a^	168.3^b^	198.2^b^	197.9^b^	44.47	0.011	0.147	0.026
IGF-2, ng/mL	14.81	13.31	11.91	13.50	4.092	0.722	0.498	0.542
T-AOC, mmol/L	0.26^b^	0.26^b^	0.31^a^	0.29^a^^b^	0.035	0.014	0.041	0.064
MDA, nmol/L	3.76	4.32	3.30	3.21	0.965	0.186	0.140	0.248
SOD, U/mL	152.6	154.2	151.6	149.1	6.85	0.675	0.319	0.483

1 TP, total protein; ALB, albumin; GLB, globulin; IgA, immunoglobulin A; IgG, immunoglobulin G; CHE, cholinesterase; GR, glutathione reductase; ALT, alanine aminotransferase; AST, aspartate aminotransferase; CRE, creatinine; BUN, blood urea nitrogen; GLU, glucose; TG, triglyceride; TC, total cholesterol; HDLC, high-density lipoprotein cholesterol; LDLC, low-density lipoprotein cholesterol; HL, hepatic lipase; GH, growth hormone; T3, triiodothyronine; T4, thyroxine; CORT, cortisol; EPI, epinephrine; INS, insulin; GC, glucagon; IGF-1, insulin-like growth factor 1; IGF-2, insulin-like growth factor 2; T-AOC, total antioxidant capacity; MDA, malondialdehyde; SOD, superoxide dismutase.

2 CON, control group; TAL, low-dose tannic acid treatment; TAM, mid-dose tannic acid treatment; TAH, high-dose tannic acid treatment.

3 SEM, standard error of means for treatments.

4 L, linear effect of the tannic acid dose; Q, quadratic effect of the tannic acid dose.

a, b, c Means within a row for treatments that do not have a common superscript differ (*P* < 0.05).

### LPS endotoxin and relevant inflammatory mediators

As is shown in Table 5, the levels of LPS in both rumen fluid and plasma, and the relevant inflammatory indicators, except the interleukin (IL)-1β, IL-6, and serumamyloid A (SAA), remained unchanged by the TA supplementation (*P* > 0.05). Further, significant increments of IL-1β (quadratic, *P* < 0.1), IL-6, and SAA (quadratic, *P* < 0.01) with the inclusion of TA in different manners were also noted.

**Table 5:** Effects of tannic acid at different doses on lipopolysaccharide endotoxin in the rumen liquid and plasma, and relevant inflammatory indicators in the serum of black goats.

**Items^1^**	**Treatments** ^2^				**SEM^3^**	***P*** **-value** ^4^		
	**CON**	**TAL**	**TAM**	**TAH**		**Dose**	**L**	**Q**
LPS-R, EU/mL	17,134	18,409	20,040	19,056	2,128.8	0.123	0.061	0.072
LPS-P, EU/mL	5,558	5,457	5,443	6,062	607.4	0.272	0.193	0.153
LBP, ng/mL	128.5	115.2	134.9	128.0	33.00	0.806	0.7744	0.937
IL-1β, pg/mL	58.5^c^	97.1^a^	72.4^b^^c^	77.4^b^	18.35	0.001	0.360	0.050
IL-2, pg/mL	973.6	1,011.1	958.9	917.2	161.16	0.823	0.474	0.661
IL-6, pg/mL	75.7^b^	106.4^a^	81.5^b^	86.3^b^	18.97	0.024	0.855	0.268
IL-8, pg/mL	127.1	144.4	139.7	161.5	32.85	0.374	0.109	0.281
TNF-α, ng/mL	115.2	140.6	116.1	121.8	21.71	0.161	0.914	0.570
IFN-γ, pg/mL	579.7	648.8	607.8	557.3	87.15	0.332	0.517	0.213
Hp, ng/mL	418.7	514.7	453.9	499.1	87.31	0.235	0.277	0.443
SAA, ng/mL	2947^b^	4185^a^	4120^a^	3418^a^^b^	816.7	0.016	0.388	0.006

1 LPS-R, lipopolysaccharide in rumen liquid; LPS-P, lipopolysaccharide in plasma; LBP, lipopolysaccharide binding protein; IL-1β, interleukin-1β; IL-2, interleukin-2; IL-6, interleukin-6; IL-8, interleukin-8; TNF-α, tumor necrosis factor alpha; IFN-γ, interferon gamma; Hp, haptoglobin; SAA, serumamyloid A.

2 CON, control group; TAL, low-dose tannic acid treatment; TAM, mid-dose tannic acid treatment; TAH, high-dose tannic acid treatment.

3 SEM, standard error of means for treatments.

4 L, linear effect of the tannic acid dose; Q, quadratic effect of the tannic acid dose.

a, b, c Means within a row for treatments that do not have a common superscript differ (*P* < 0.05).

### Taxonomic annotation of rumen bacterial community

An average of 4,262 ± 761 sequences and an average of 179 ± 138 OTUs per sample were respectively acquired through the quality filtration (Supplementary Table S1). In sum, 15 bacterial phyla were detected across all the samples (Supplementary Table S2). *Bacteroidetes* (49.6 ± 11.36 %), *Firmicutes* (32.4 ± 10.40 %), and *Proteobacteria* (9.0 ± 11.76 %) were the top three dominant phyla, accounting for 91.0 ± 4.22 % of the whole bacterial microbiome across all the samples (Supplementary Figure S1). A total of 110 bacterial genera were totally identified, and the rumen bacterial community was primarily predominated by *Prevotella_1* (17.3 ± 8.33 %), *uncultured_bacterium_f_Muribaculaceae* (16.9 ± 9.61 %), *Succiniclasticum* (8.6 ± 4.66 %), and *Rikenellaceae_RC9_gut_group* (5.0 ± 3.28 %) (Supplementary Figure S2). At the species level, 128 bacterial species were annotated altogether, with *uncultured_bacterium_f_Muribaculaceae* (16.9 ± 9.61%), *uncultured_bacterium_g_Prevotella_1* (16.9 ± 8.39%), *uncultured_bacterium_g_Succiniclasticum* (7.9 ± 5.14%), and *uncultured_bacterium_g_Rikenellaceae_RC9_gut_group* (5.0 ± 3.24%) basically being the most dominant (Supplementary Figure S3). As was depicted by the Venn diagram (Supplementary Figure S4), 362, 651, 388, and 381 OTUs were obtained in the CON, TAL, TAM, and TAH, with the unique OTU number of 7, 6, 9, and 12, respectively. Besides, the majority of those exclusive OTUs of each treatment at the phylum level were classified as either *Bacteroidetes, Firmicutes*, or *Proteobacteria* (Supplementary Table S3).

### Diversity of rumen bacterial community

**Figure 1:**
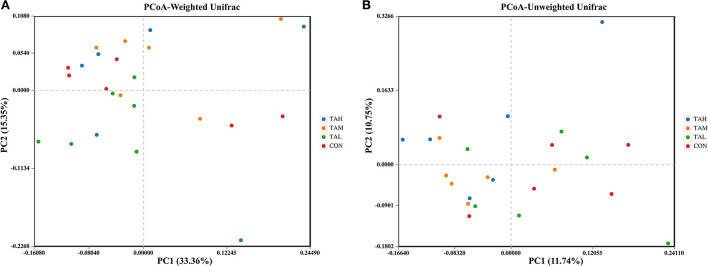
The principal coordinate analysis (PCoA) plots on rumen bacterial community structure across treatments. **(A)** PCoA based on the weighted Unifrac matrix. **(B)** PCoA based on the unweighted Unifrac matrix.

The Alpha diversity indexes of ACE, Chao 1, Shannon, and Simpson for the bacterial microflora in the rumen fluid of black goats were all unaffected (*P* > 0.05) by the addition of TA at different doses (Table 6). For the Beta diversity of rumen bacterial microbiome across treatments, no group-dependent clustering of the bacterial community was illustrated through the analysis based on either the weighted or unweighted Unifrac matrix (Figure 1).

**Table 6:** Effects of tannic acid at different doses on Alpha diversity indices of the bacterial community in the rumen fluid of black goats.

**Items**	**Treatments** ^a^				**SEM^b^**	***P*** **-value** ^c^		
	**CON**	**TAL**	**TAM**	**TAH**		**Dose**	**L**	**Q**
Ace	204	219	241	222	36.3	0.401	0.265	0.287
Chao 1	198	213	241	224	36.3	0.224	0.113	0.159
Shannon	4.95	4.95	5.25	4.93	0.646	0.825	0.836	0.827
Simpson	0.92	0.92	0.91	0.90	0.047	0.896	0.509	0.799

a CON, control group; TAL, low-dose tannic acid treatment; TAM, mid-dose tannic acid treatment; TAH, high-dose tannic acid treatment.

b SEM, standard error of means for treatments.

c L, linear effect of the tannic acid dose; Q, quadratic effect of the tannic acid dose.

### Differential rumen bacterial taxa within treatments

**Figure 2:**
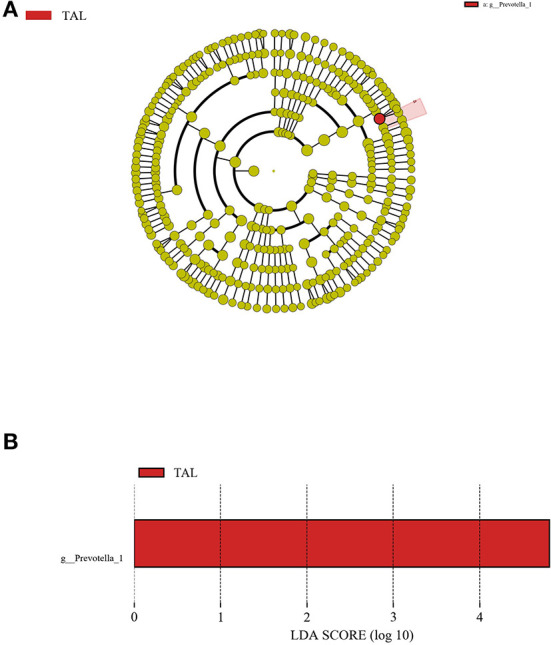
The LDA effect size (LEfSe) analysis of bacterial taxa across treatments. **(A)** Cladogram displays significantly enriched bacterial taxa (from the phylum to the species level). Red: taxa abundant in the TAL treatment. **(B)** Bar chart displays LDA scores across treatments. The LDA scores represented the difference in relative abundance with exponent fold change of 10 across treatments. Significant differences are defined as *P* < 0.05 and LDA score > 4.0.

As was demonstrated through the LEfSe analysis from the phylum level to the species level, the genus *Prevotella_1* was the only one detected differential bacterial taxa which was significantly (*P* < 0.05) enriched in TAL with a LDA score > 4.0 when compared to other treatments (Figure 2).

### Function prediction of rumen bacterial microbiota

**Figure 3:**
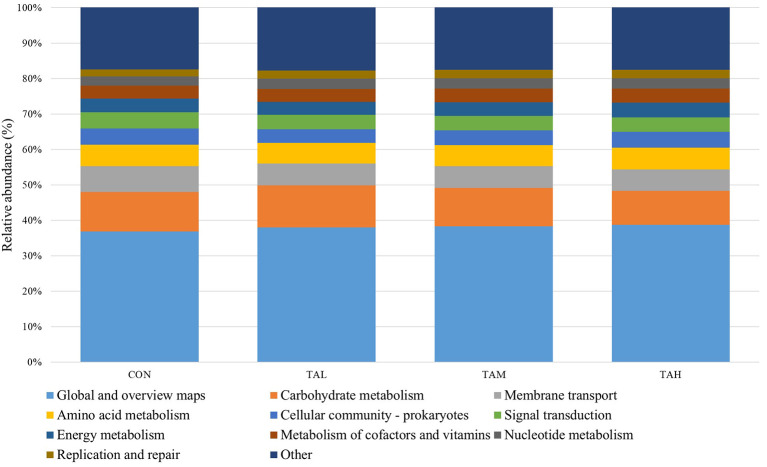
The top ten annotated KEGG pathways (at level 2) across treatments based on Tax4Fun function prediction.

The function estimation based on Tax4Fun revealed that the global and overview maps, carbohydrate metabolism, membrane transport, and amino acid metabolism were identified with the highest KEEG orthologs (KO) abundances amongst the top 10 KEGG pathways (Figure 3). Further, no significant (*P* > 0.05) difference in the relative frequencies of those annotated KEGG pathways across treatments was observed through the *t*-test analysis (Supplementary Table S4).

## Discussion

Tannins are usually considered to exert adverse impacts on the feed intake of ruminants, due to the relevant low palatability, reduced digesta evacuation rate out of the rumen, and poisonousness (35). Nonetheless, no influence of either CTs or HTs on ruminants feed intake has been observed in a series of previous research. Henke et al. (14) reported that the inclusion of quebracho CT at neither 1.5 % nor 3.0% of DM affected the DM and OM intake of Holstein dairy cattle, whilst Liu et al. (3) found no effect on the DMI by supplementing either 1.0 % or 3.0 % chestnut tannins (high in HT) in the diet of sheep. Further, the DMI of beef cattle fed a high-forage ration remained unaffected by either the single addition of chestnut HT (0.25 or 1.5 % of DM), or the equal mixture of chestnut HT and quebracho CT (0.125 or 0.75 % of DM of each) (8). Moreover, it was revealed that none of the DMI of Simmental cattle was influenced by adding 1.69 % TA in the diet containing either 11.1 or 13.6 % CP (2). Similarly, the TA supplementation at different doses did not alter the DMI of goats in the present trial. This phenomenon could be explained by the tolerance for tannins of goats, especially the presence of tannin-binding proteins in the saliva of goats (36).

Various declines in the nutrient digestion of cattle in response to the dietary supplementation of CTs or HTs have been demonstrated by precedent studies. In comparison, the influence of TA on the nutrient digestibility in goats has been rarely reported. Zhang et al. (11) reported that the apparent digestibility of DM, NDF, ADF, and CP were reduced by the supplementation of *Acacia mangium* CT at 3 % of DM, whereas adding 3 % valonia HT merely decreased CP digestibility in dairy cows. Yang et al. (5) found that the TA inclusion at 0.65 % and 1.3 % of DM only decreased CP digestion, while adding 2.6 % TA lowered the digestibility of DM, OM, and CP in beef cattle. Subsequently, it was marked that supplementing 1.69 % TA reduced the digestion of DM, OM, and CP in beef cattle (27). In this study, only the CP digestibility of black goats was reduced by adding 0.6 and 0.9 % TA, which might be associated with the relatively low proportions of TA compared with the above studies, as well as the discrepancies in tannin types, diets, and animals. The reduction in the apparent total tract digestibility of CP by TA could be ascribed to the affinity of TA for dietary proteins and the incomplete disassociation of the complexes within abomasum (37, 38), and indicates that the tolerance for TA of goats might be somewhat limited.

Despite the fact that CP digestion was suppressed by adding TA, the ADG and FCR of goats both remained unchanged in the current study. Likewise, Aboagye et al. (8) found that neither the sole inclusion of HT nor the even combination of HT and CT changed the ADG and gain to feed ratio of beef cattle. Besides, no influence of chestnut HT supplemented at 1.0 or 3.0 % of DM on the ADG and FCR was shown in a study on sheep (3). Although tannins could raise the amount of RUP through their protein-binding capacity, they can also restrain the protein synthesis by ruminal microbes, hence the MP that can be utilized for growth might not be absolutely improved by tannins supplementation (12).

Previous investigations have found that the level of ruminal NH_3_-N in beef cattle was lowered by TA addition at different doses (2, 5), which is consistent with the significant drop of the NH_3_-N concentration in TAM and TAH of this study. This response might be contributed to the binding capacity of TA with feed proteins and thence the protection of proteins against the degradation by ruminal microbes (39). As for the ruminal VFA, declines in TVFA concentration, and the molar percentages of valerate and isovalerate by 1.69 % TA addition were previously marked in beef cattle (2). In contrary, dramatic increment of TVFA in TAM and TAH was noticed in the present trial, along with the significant rise of valerate in TAM. This result seems contradictory to the responses in DMI and nutrient digestibility of goats in the current experiment. However, since TA could lower the digesta evacuation rate out of the rumen (35), the VFA clearance might be reduced and thereby result in the rise of ruminal VFA (40). Further investigations are required to examine this assumption. In addition, individual variations in ruminal TVFA and other parameters measured in this study were noted, similar to our precedent findings on the establishment and development of rumen microbiome in black goats (32, 33).

In the current trial, TA exerted influences on the blood biochemical and physiological indexes despite its possible degradation in the gastrointestinal tract (8). The blood concentrations of ALT and AST are normally considered as indicators of liver function (41). As was reported by Yang et al. (5), none of the ALT and AST in the plasma of beef cattle was influenced by TA addition at 0.65, 1.3, or 2.6 % of DM. Nevertheless, the increment of serum ALT by supplementing 0.6% of DM was marked in this study, implying that TA might cause the hepatic injury in goats (41). Besides, Yang et al. (5) also found that the inclusion of TA at different doses did not change the plasma concentrations of total protein (TP) and albumin (ALB). By contrast, in the present trial, the abatements of GLB, IgG, CRE, GH, and IGF-1 to different extents were observed when TA was supplemented, which could be linked to the suppressed CP digestibility described above and the possibly consequent protein deficiency, implying that TA addition possibly exerted negative influences on the immune function (42), muscle mass (43), and somatotropic system (44) of goats. In the present experiment, the dietary CP level (7.39% of DM) was relatively low, probably resulting in the adverse effects of TA even supplemented at low doses. It could be thence assumed that the negative influences of TA on goats might be alleviated by raising CP concentration in the diet, and more studies should be performed to examine this hypothesis.

It was noteworthy that the TG concentration in the blood of goats was enhanced by the TA supplementation in the current study, this phenomenon suggested that the endogenous TG synthesis primarily in liver could be elevated, and could have relation to the above-mentioned rise in ruminal TVFA which might raise the VFA delivery to liver (45, 46). Besides, the reduction of serum HDLC by adding TA was also observed in this study, indicating the potentially increased oxidative stress of goats (47). Furthermore, a recent research found that the serum HDLC level can serve as a biomarker for the trunk muscle volume and function (48), making the HDLC decrement in line with the CRE decline of the present trial. In addition, the reverse responses of CORT (increase) and CHE (decrease) to TA supplementation were noticed, supported by a prior study in which CHE inhibition induced the elevation of CORT level (49). Further, the higher level of CORT in TAL could reflect an escalated degree of stress (50), and the lower GR concentration in TAH might imply the decrease of antioxidative ability (51). Adding TA at 0.6% of DM raised the total antioxidant T-AOC in this trial, which was consistent with the finding of Yang et al. (5) that the plasma T-AOC was increased by the inclusion of 0.65, 1.3, and 2.6% TA. However, given the present alterations in HDLC, CORT, and GR, the enhancement of T-AOC could be a compensatory response of goats to the escalation of oxidative stress (52). The inconsistency in the effects of TA on oxidation status between previous investigations (16–18) and the current study might stem from the disparities in TA source, dosage, diet, and animal, and necessitates further research to be explained.

Upon the translocation of LPS endotoxin from rumen into the peripheral blood, the secretion of a series of pro-inflammatory cytokines and systemic inflammation would be provoked (29, 53). No impact on the LPS level in rumen fluid or plasma by TA addition was illustrated by the present trial. Nevertheless, supplementing TA raised the concentrations of the inflammatory cytokines including IL-1β and IL-6, and the relevant acute phase protein SAA to different extents, suggesting that TA could trigger inflammatory reaction in goats (54, 55). More studies are needed to disclose the mechanisms of TA-induced inflammation in goats.

At the phylum level, the bacterial community in rumen fluid was successively dominated by *Bacteroidetes, Firmicutes*, and *Proteobacteria* in the current trial, which was in agreement with the discovery on the ruling bacterial phyla within ruminal microbiota in precedent studies including our previous reports (23, 25, 32, 56, 57). Besides, the predominance of the genera *Prevotella_1* and *Rikenellaceae_RC9_gut_group* in the rumen bacterial populations was observed, also being supported by prior investigations in which the high relative abundances of these two taxa were present in the rumen (58, 59). As a major proteolytic bacteria amongst the rumen microorganisms, the *Prevotella* species can degrade protein into ammonia (60). Therefore, the enrichment of *Prevotella_1* in TAL might attenuate the adverse impacts of TA on the protein fermentation in rumen, and this inference is consistent with the unaffected NH_3_-N concentration in TAL compared to CON. Moreover, the negative correlation between the ruminal *Prevotella_1* abundance and amino acid metabolism and energy metabolism in goats was demonstrated by a recent study (61). This finding implies that a potential relationship might exist between the enrichment of *Prevotella_1* and the reduction of GLB, IgG, GH, and IGF-1 in TAL goats, which requires further explorations to be verified.

In the present experiment, the Alpha and Beta diversity of the bacterial community, as well as relative portions of most bacterial taxa at different levels were unchanged by supplementing goats with TA, indicating the endurance and stability of the entire ruminal microflora (2). In addition, the contradiction between the results of function prediction for bacterial populations and rumen fermentation traits across treatments in the current trial might be explained by the imperfection of Tax4Fun analysis, differences between the liquid- and solid-phase bacterial community, and discrepancy between metagenomics analysis and actual metabolisms of the ruminal microbiota (23).

## Conclusion

In this study, despite the unaffected growth performance, the supplementation of TA at different doses generally exerted negative influences on the CP digestion, as well as the physiological, antioxidative and immunological functions to different extents. Therefore, more investigations should be conducted to determine the safe dosage of TA added to different diets for goats, and reveal the mechanisms concerning the impacts of TA on goats.

## Data availability statement

The datasets presented in this study can be found in online repositories. The names of the repository/repositories and accession number(s) can be found below: https://www.ncbi.nlm.nih.gov/, PRJNA847688.

## Ethics statement

The animal study was reviewed and approved by Animal Care Committee, College of Animal Science and Technology, and Hunan Agricultural University.

## Author contributions

ZW, JH, FW, and WS designed the research. ZW, LY, LL, XL, FW, WS, ST, ZT, and YY conducted the research. ZW and LY analyzed the data. ZW wrote the paper. All authors approved the final manuscript.

## Funding

The present work was supported by the Hunan Provincial Education Department (Grant No. 19B257), Hunan Herbivores Industry Technological System, Hunan Provincial Natural Science Foundation (Grant No. 2019JJ50279), China Agriculture Research System of MOF and MARA (Grant No. CARS-37), and National Natural Science Foundation of China (Grant No. 31772633).

## Conflict of interest

Author YY was employed by Jiurui Biology and Chemistry Co., Ltd.

The remaining authors declare that the research was conducted in the absence of any commercial or financial relationships that could be construed as a potential conflict of interest.

## Publisher's note

All claims expressed in this article are solely those of the authors and do not necessarily represent those of their affiliated organizations, or those of the publisher, the editors and the reviewers. Any product that may be evaluated in this article, or claim that may be made by its manufacturer, is not guaranteed or endorsed by the publisher.

## References

[1] Aboagye IA Oba M Koenig KM Zhao GY Beauchemin KA. Use of gallic acid and hydrolyzable tannins to reduce methane emission and nitrogen excretion in beef cattle fed a diet containing alfalfa silage. J Anim Sci. (2019) 97:2230–44. 10.1093/jas/skz101 30906949 PMC6488321

[2] Zhou K Bao Y Zhao G. Effects of dietary crude protein and tannic acid on rumen fermentation, rumen microbiota and nutrient digestion in beef cattle. Arch Anim Nutr. (2019) 73:30–43. 10.1080/1745039X.2018.1545502 30512985

[3] Liu H Vaddella V Zhou D. Effects of chestnut tannins and coconut oil on growth performance, methane emission, ruminal fermentation, and microbial populations in sheep. J Dairy Sci. (2011) 94:6069–77. 10.3168/jds.2011-4508 22118094

[4] Patra AK Saxena J. Exploitation of dietary tannins to improve rumen metabolism and ruminant nutrition. J Sci Food Agric. (2011) 91:24–37. 10.1002/jsfa.4152 20815041

[5] Yang K Wei C Zhao GY Xu ZW Lin SX. Effects of dietary supplementing tannic acid in the ration of beef cattle on rumen fermentation, methane emission, microbial flora and nutrient digestibility. J Anim Physiol Anim Nutr. (2017) 101:302–10. 10.1111/jpn.12531 27272696

[6] Majewska MP Miltko R Bełzecki G Kedzierska A Kowalik B. Comparison of the effect of synthetic (Tannic Acid) or Natural (Oak Bark Extract) hydrolysable tannins addition on fatty acid profile in the rumen of sheep. Animals. (2022) 12:699. 10.3390/ani12060699 35327095 PMC8944490

[7] Vasta V Daghio M Cappucci A Buccioni A Serra A Viti C. Invited review: Plant polyphenols and rumen microbiota responsible for fatty acid biohydrogenation, fiber digestion, and methane emission: Experimental evidence and methodological approaches. J Dairy Sci. (2019) 102:3781–804. 10.3168/jds.2018-14985 30904293

[8] Aboagye IA Oba M Castillo AR Koenig KM Iwaasa AD Beauchemin KA. Effects of hydrolyzable tannin with or without condensed tannin on methane emissions, nitrogen use, and performance of beef cattle fed a high-forage diet. J Anim Sci. (2018) 96:5276–86. 10.1093/jas/sky404.406 30169710 PMC6276562

[9] Menci R Coppa M Torrent A Natalello A Valenti B Luciano G. Effects of two tannin extracts at different doses in interaction with a green or dry forage substrate on in vitro rumen fermentation and biohydrogenation. Anim Feed Sci Technol. (2021) 278:114977. 10.1016/j.anifeedsci.2021.11497734835430

[10] Sarnataro C Spanghero M. In vitro rumen fermentation of feed substrates added with chestnut tannins or an extract from Stevia rebaudiana Bertoni. Anim Nutr. (2020) 6:54–60. 10.1016/j.aninu.2019.11.009 32211529 PMC7082680

[11] Zhang J Xu X Cao Z Wang Y Yang H Azarfar A. Effect of different tannin sources on nutrient intake, digestibility, performance, nitrogen utilization, and blood parameters in dairy cows. Animals. (2019) 9:507. 10.3390/ani9080507 31370306 PMC6719915

[12] MacAdam J Villalba J. Beneficial effects of temperate forage legumes that contain condensed tannins. Agriculture. (2015) 5:475–91. 10.3390/agriculture5030475

[13] Mezzomo R Paulino PVR Detmann E Valadares Filho SC Paulino MF Monnerat JPIS. Influence of condensed tannin on intake, digestibility, and efficiency of protein utilization in beef steers fed high concentrate diet. Livest Sci. (2011) 141:1–11. 10.1016/j.livsci.2011.04.004

[14] Henke A Dickhoefer U Westreicher-Kristen E Knappstein K Molkentin J Hasler M. Effect of dietary Quebracho tannin extract on feed intake, digestibility, excretion of urinary purine derivatives and milk production in dairy cows. Arch Anim Nutr. (2017) 71:37–53. 10.1080/1745039X.2016.1250541 27830586

[15] Zhou K Bao Y Zhao G. Effects of dietary crude protein and tannic acid on nitrogen excretion, urinary nitrogenous composition and urine nitrous oxide emissions in beef cattle. J Anim Physiol Anim Nutr. (2019) 103:1675–83. 10.1111/jpn.13186 31469196

[16] Lou W Chen Y Ma H Liang G Liu B. Antioxidant and α-amylase inhibitory activities of tannic acid. J Food Sci Technol. (2018) 55:3640–6. 10.1007/s13197-018-3292-x 30150823 PMC6098770

[17] Sultana S Sehrawat A. Abrogation of thioacetamide-induced biochemical events of hepatic tumor promotion stage by tannic acid in wistar rats. J Environ Pathol Toxicol Oncol. (2007) 26:9–20. 10.1615/JEnvironPatholToxicolOncol.v26.i1.20 17725526

[18] Ugur Calis I Turgut Cosan D Saydam F Kerem Kolac U Soyocak A Kurt H. The effects of monosodium glutamate and tannic acid on adult rats. Iran Red Crescent Med J. (2016) 18:e37912. 10.5812/ircmj.37912 28184327 PMC5291937

[19] dos Santos JDC Saraiva EP Gonzaga Neto S Saraiva CAS da Pinheiro CA de Fonsêca VFC. Feeding behavior of lactating dairy cattle fed sorghum-based diets and increasing levels of tannic acid. Agriculture. (2021) 11:172. 10.3390/agriculture11020172

[20] Díaz Carrasco JM Cabral C Redondo LM Pin Viso ND Colombatto D Farber MD. Impact of chestnut and quebracho tannins on rumen microbiota of bovines. Biomed Res Int. (2017) 2017:1–11. 10.1155/2017/9610810 29445749 PMC5763072

[21] Min BR Wright C Ho P. The effect of phytochemical tannins-containing diet on rumen fermentation characteristics and microbial diversity dynamics in goats using 16S rDNA amplicon pyrosequencing. Agric Food Anal Bacteriol. (2014) 4:195–211. 10.1155/2014/141909 24669219 PMC3941959

[22] He Q Kwok LY Xi X Zhong Z Ma T Xu H. The meconium microbiota shares more features with the amniotic fluid microbiota than the maternal fecal and vaginal microbiota. Gut Microbes. (2020) 12:1794266. 10.1080/19490976.2020.1794266 32744162 PMC7524391

[23] Wang Z Yu Y Li X Xiao H Zhang P Shen W. Fermented soybean meal replacement in the diet of lactating holstein dairy cows: modulated rumen fermentation and ruminal microflora. Front Microbiol. (2021) 12:625857. 10.3389/fmicb.2021.625857 33584627 PMC7879537

[24] Shen JS Chai Z Song LJ Liu JX Wu YM. Insertion depth of oral stomach tubes may affect the fermentation parameters of ruminal fluid collected in dairy cows. J Dairy Sci. (2012) 95:5978–84. 10.3168/jds.2012-5499 22921624

[25] Wang Z Yang DS Li XY Yu YN Yong LY Zhang PH. Modulation of rumen fermentation and microbial community through increasing dietary cation–anion difference in Chinese Holstein dairy cows under heat stress conditions. J Appl Microbiol. (2020). 10.1111/jam.14812 32757409

[26] Wang Z Li XY Yu YN Yang LY Zhang PH He JH. Enhancing dietary cation-anion difference reshaped the distribution of endotoxin across different biofluids and influenced inflammatory response in dairy cows exposed to heat stress. Anim Feed Sci Technol. (2020) 262:114444. 10.1016/j.anifeedsci.2020.114444

[27] Tang SX He Y Zhang PH Jiao JZ Han XF Yan QX. Nutrient digestion, rumen fermentation and performance as ramie (Boehmeria nivea) is increased in the diets of goats. Anim Feed Sci Technol. (2019) 247:15–22. 10.1016/j.anifeedsci.2018.10.013

[28] Wang Z He Z Beauchemin KA Tang S Zhou C Han X. Comparison of two live Bacillus species as feed additives for improving in vitro fermentation of cereal straws: Live Bacillus feed addition. Anim Sci J. (2016) 87:27–36. 10.1111/asj.12346 26611805

[29] Wang Z Zhang L Li Z Yu Y Yang L Zhang P. Alterations of endotoxin distribution across different biofluids and relevant inflammatory responses by supplementing L-theanine in dairy cows during heat stress. Anim Nutr. (2021) 7:1253–7. 10.1016/j.aninu.2021.03.012 34786498 PMC8566959

[30] Guzmán JL Perez-Ecija A Zarazaga LA Martín-García AI Horcada A Delgado-Pertíñez M. Using dried orange pulp in the diet of dairy goats: effects on milk yield and composition and blood parameters of dams and growth performance and carcass quality of kids. Animal. (2020) 14:2212–20. 10.1017/S1751731120000932 32367792

[31] Yu Z Morrison M. Improved extraction of PCR-quality community DNA from digesta and fecal samples. Biotechniques. (2004) 36:808–12. 10.2144/04365ST04 15152600

[32] Wang Z Elekwachi C Jiao J Wang M Tang S Zhou C. Changes in metabolically active bacterial community during rumen development, and their alteration by rhubarb root powder revealed by 16S rRNA amplicon sequencing. Front Microbiol. (2017) 8:159. 10.3389/fmicb.2017.00159 28223972 PMC5293741

[33] Wang Z Elekwachi CO Jiao J Wang M Tang S Zhou C. Investigation and manipulation of metabolically active methanogen community composition during rumen development in black goats. Sci Rep. (2017) 7:422. 10.1038/s41598-017-00500-5 28341835 PMC5428682

[34] Aßhauer KP Wemheuer B Daniel R Meinicke P. Tax4Fun: predicting functional profiles from metagenomic 16S rRNA data. Bioinformatics. (2015) 31:2882–4. 10.1093/bioinformatics/btv287 25957349 PMC4547618

[35] Lamy E Rawel H Schweigert FJ. Capela e Silva F Ferreira A Costa AR. The effect of tannins on mediterranean ruminant ingestive behavior: the role of the oral cavity. Molecules. (2011) 16:2766–84. 10.3390/molecules16042766 21441875 PMC6260606

[36] Schmitt MH Ward D Shrader AM. Salivary tannin-binding proteins: a foraging advantage for goats? Livest Sci. (2020) 234:103974. 10.1016/j.livsci.2020.103974

[37] Bravo L. Polyphenols: Chemistry, Dietary Sources, Metabolism, and Nutritional Significance. Nutr Rev. (2009) 56:317–33. 10.1111/j.1753-4887.1998.tb01670.x 9838798

[38] Smith AH Zoetendal E Mackie RI. Bacterial mechanisms to overcome inhibitory effects of dietary tannins. Microb Ecol. (2005) 50:197–205. 10.1007/s00248-004-0180-x 16222487

[39] Getachew G Pittroff W Putnam DH Dandekar A Goyal S DePeters EJ. The influence of addition of gallic acid, tannic acid, or quebracho tannins to alfalfa hay on in vitro rumen fermentation and microbial protein synthesis. Anim Feed Sci Technol. (2008) 140:444–61. 10.1016/j.anifeedsci.2007.03.011

[40] Bannink A France J Lopez S Gerrits WJJ Kebreab E Tamminga S. Modelling the implications of feeding strategy on rumen fermentation and functioning of the rumen wall. Anim Feed Sci Technol. (2008) 143:3–26. 10.1016/j.anifeedsci.2007.05.002

[41] Magistrelli D Aufy AA Pinotti L Rosi F. Analysis of weaning-induced stress in Saanen goat kids: Weaning-induced stress in goat kids. J Anim Physiol Anim Nutr. (2013) 97:732–9. 10.1111/j.1439-0396.2012.01315.x 22715986

[42] Furman-Fratczak K Rzasa A Stefaniak T. The influence of colostral immunoglobulin concentration in heifer calves' serum on their health and growth. J Dairy Sci. (2011) 94:5536–43. 10.3168/jds.2010-3253 22032377

[43] Bruce HL Hewavitharana AK Hunter RA. Creatinine and pseudouridine in plasma and urine from Brahman-cross steers fed a low, medium or high plane of nutrition. Livest Sci. (2008) 119:95–101. 10.1016/j.livsci.2008.03.006

[44] López-Flores NM Meza-Herrera CA Galán-Soldevilla C Bautista-Rodriguez DA Veliz-Deras FG Arellano-Rodriguez G. The key role of targeted betacarotene supplementation on endocrine and reproductive outcomes in goats: follicular development, ovulation rate and the GH-IGF-1 axis. Small Rumin Res. (2018) 163:29–33. 10.1016/j.smallrumres.2017.09.009

[45] Andrade GP de Carvalho FFR de Batista ÂMV Pessoa RAS Costa CA da Cardoso DB. Evaluation of crude glycerin as a partial substitute of corn grain in growing diets for lambs. Small Rumin Res. (2018) 165:41–7. 10.1016/j.smallrumres.2018.06.002

[46] Grummer RR. Etiology of lipid-related metabolic disorders in periparturient dairy cows. J Dairy Sci. (1993) 76:3882–96. 10.3168/jds.S0022-0302(93)77729-2 8132893

[47] Karabacak M Varol E Kahraman F Ozaydin M Türkdogan AK Ersoy IH. Low High-density lipoprotein cholesterol is characterized by elevated oxidative stress. Angiology. (2014) 65:927–31. 10.1177/0003319713512173 24280265

[48] Shirahata T Sato H Yogi S Inoue K Niitsu M Miyazawa H. Possible association of high-density lipoprotein cholesterol levels with trunk muscle deficits and decrease in energy expenditure in patients with or at risk for COPD: A pilot study. Respir Investig. (2022) 60:720–4. 10.1016/j.resinv.2022.06.005 35821189

[49] Cobilinschi C. Endocrine disturbances induced by low-dose organophosphate exposure in male wistar rats. Acta Endo (Buc). (2021) 17:177–85. 10.4183/aeb.2021.177 34925565 PMC8665251

[50] Aghamiri SM Samimi AS Hajian M Samimi AM Oroumieh A. Effect of xylazine, detomidine, medetomidine and dexmedetomidine during laparoscopic SCNT embryo transfer on pregnancy rate and some physiological variables in goats. BMC Vet Res. (2022) 18:98. 10.1186/s12917-022-03194-8 35292035 PMC8922821

[51] Sun ZH He ZX Zhang QL Tan ZL Han XF Tang SX. Effects of protein and/or energy restriction for six weeks on antioxidation capacity of plasma and gastrointestinal epithelial tissues of weaned kids. Livest Sci. (2012) 149:232–41. 10.1016/j.livsci.2012.07.014

[52] Bernabucci U Ronchi B Lacetera N Nardone A. Markers of oxidative status in plasma and erythrocytes of transition dairy cows during hot season. J Dairy Sci. (2002) 85:2173–9. 10.3168/jds.S0022-0302(02)74296-3 12362449

[53] Plaizier JC Khafipour E Li S Gozho GN Krause DO. Subacute ruminal acidosis (SARA), endotoxins and health consequences. Anim Feed Sci Technol. (2012) 172:9–21. 10.1016/j.anifeedsci.2011.12.004

[54] Wang D Gao Q Zhao G Kan Z Wang X Wang H. Protective effect and mechanism of theanine on lipopolysaccharide-induced inflammation and acute liver injury in mice. J Agric Food Chem. (2018) 66:7674–83. 10.1021/acs.jafc.8b02293 29969892

[55] Ye H Liu J Feng P Zhu W Mao S. Grain-rich diets altered the colonic fermentation and mucosa-associated bacterial communities and induced mucosal injuries in goats. Sci Rep. (2016) 6:20329. 10.1038/srep20329 26841945 PMC4740883

[56] Jami E Israel A Kotser A Mizrahi I. Exploring the bovine rumen bacterial community from birth to adulthood. ISME J. (2013) 7:1069–79. 10.1038/ismej.2013.2 23426008 PMC3660679

[57] Rey M Enjalbert F Combes S Cauquil L Bouchez O Monteils V. Establishment of ruminal bacterial community in dairy calves from birth to weaning is sequential. J Appl Microbiol. (2014) 116:245–57. 10.1111/jam.12405 24279326

[58] Cui X Wang Z Tan Y Chang S Zheng H Wang H. Selenium yeast dietary supplement affects rumen bacterial population dynamics and fermentation parameters of tibetan sheep (Ovis aries) in alpine meadow. Front Microbiol. (2021) 12:663945. 10.3389/fmicb.2021.663945 34276597 PMC8283570

[59] Wang H He Y Li H Wu F Qiu Q Niu W. Rumen fermentation, intramuscular fat fatty acid profiles and related rumen bacterial populations of Holstein bulls fed diets with different energy levels. Appl Microbiol Biotechnol. (2019) 103:4931–42. 10.1007/s00253-019-09839-3 31020378

[60] He Y Yu Z Qiu Q Shao T Niu W Xia C. Effects of dietary protein levels and calcium salts of long-chain fatty acids on nitrogen mobilization, rumen microbiota and plasma fatty acid composition in Holstein bulls. Anim Feed Sci Technol. (2018) 246:1–10. 10.1016/j.anifeedsci.2018.09.019

[61] Xue B Wu M Yue S Hu A Li X Hong Q. Changes in rumen bacterial community induced by the dietary physically effective neutral detergent fiber levels in goat diets. Front Microbiol. (2022) 13:820509. 10.3389/fmicb.2022.820509 35479630 PMC9035740

