# Supplementary figures and images for: Tannic acid reduced apparent protein digestibility and induced oxidative stress and inflammatory response without altering growth performance and ruminal microbiota diversity of Xiangdong black goats
Source: Front Vet Sci. 2022 Sep 8;9:1004841. doi: 10.3389/fvets.2022.1004841 (PMC9516568; doi:10.3389/fvets.2022.1004841)

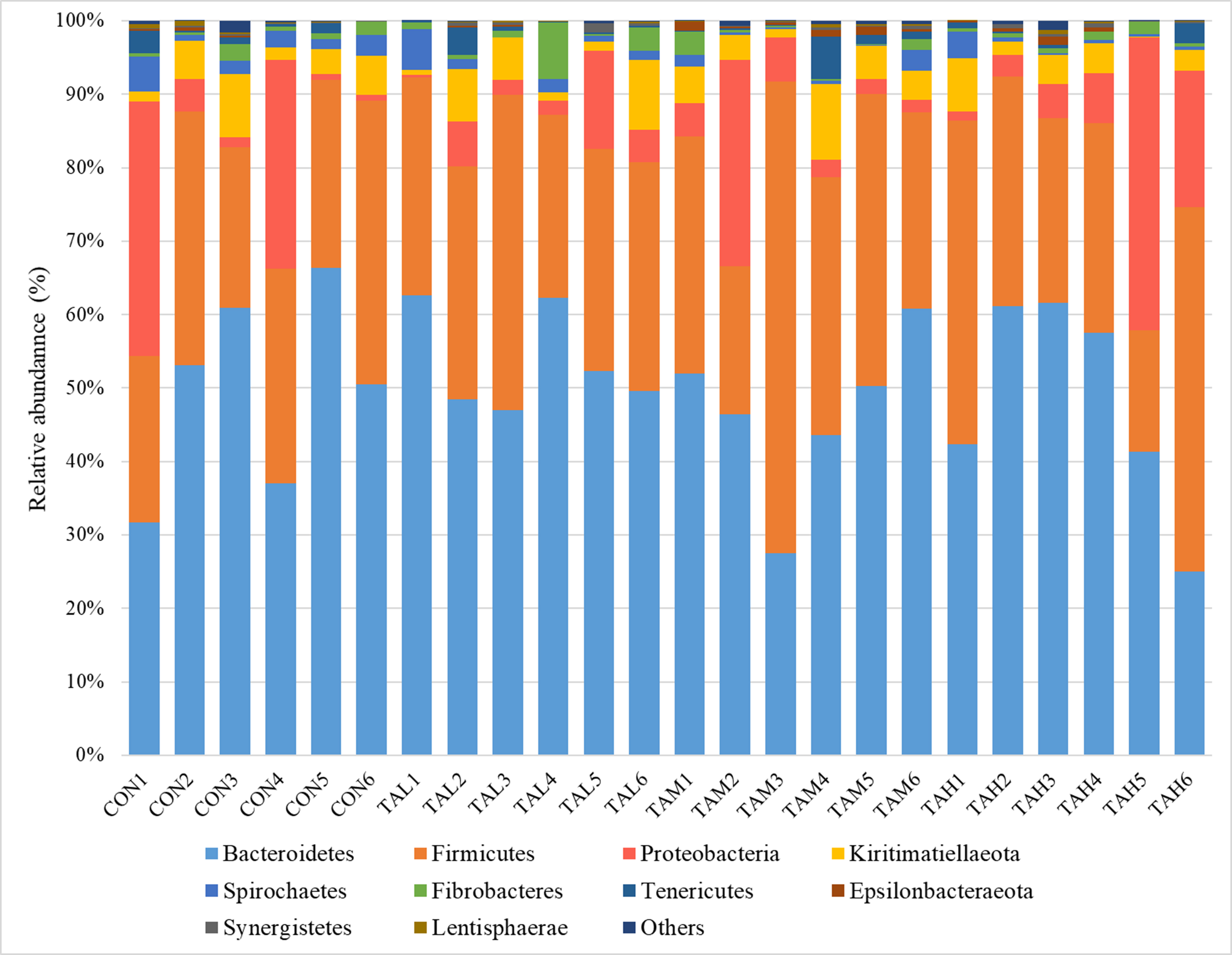

Supplement: Supplementary file 5 [file Image_1.jpg]

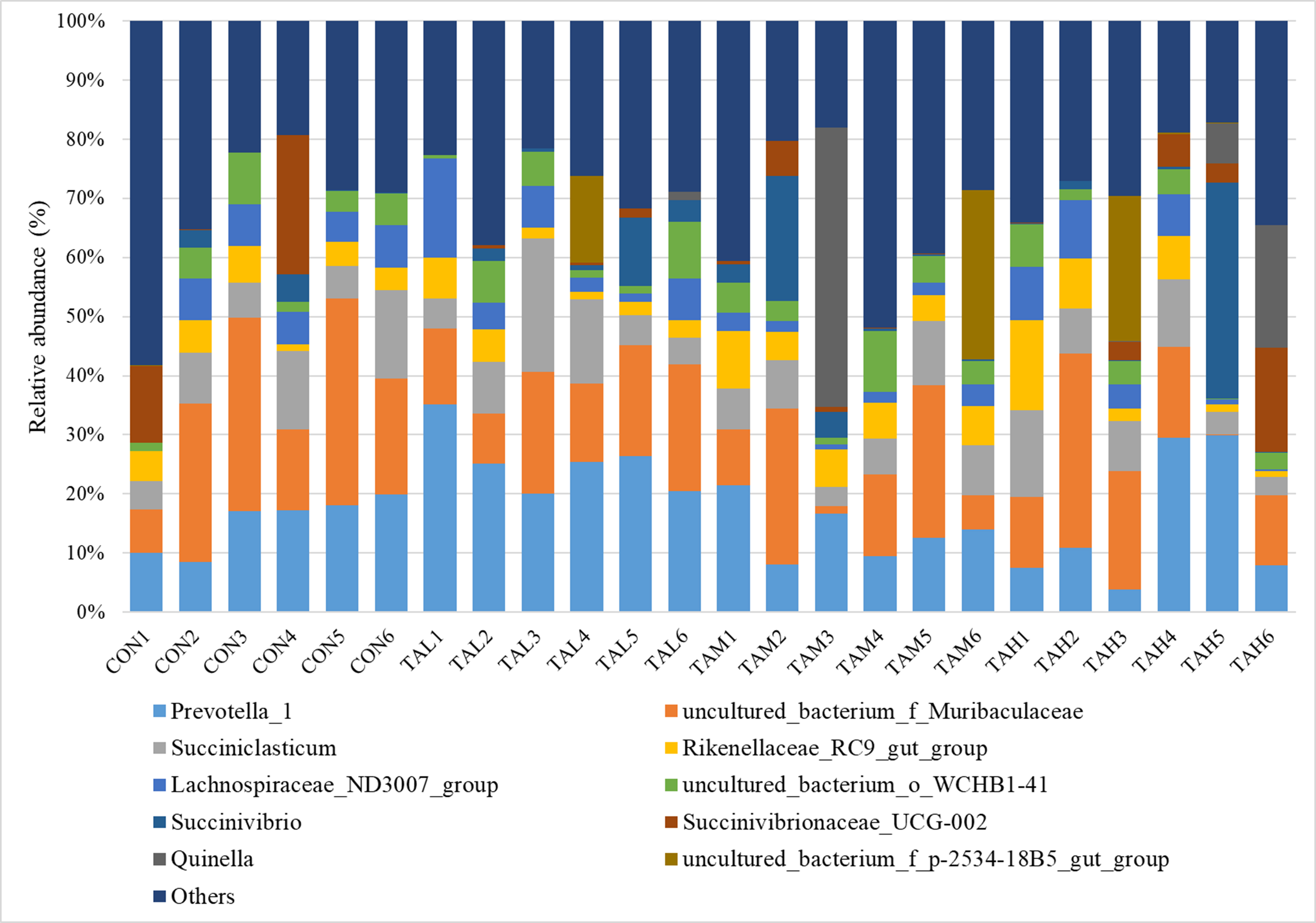

Supplement: Supplementary file 6 [file Image_2.jpg]

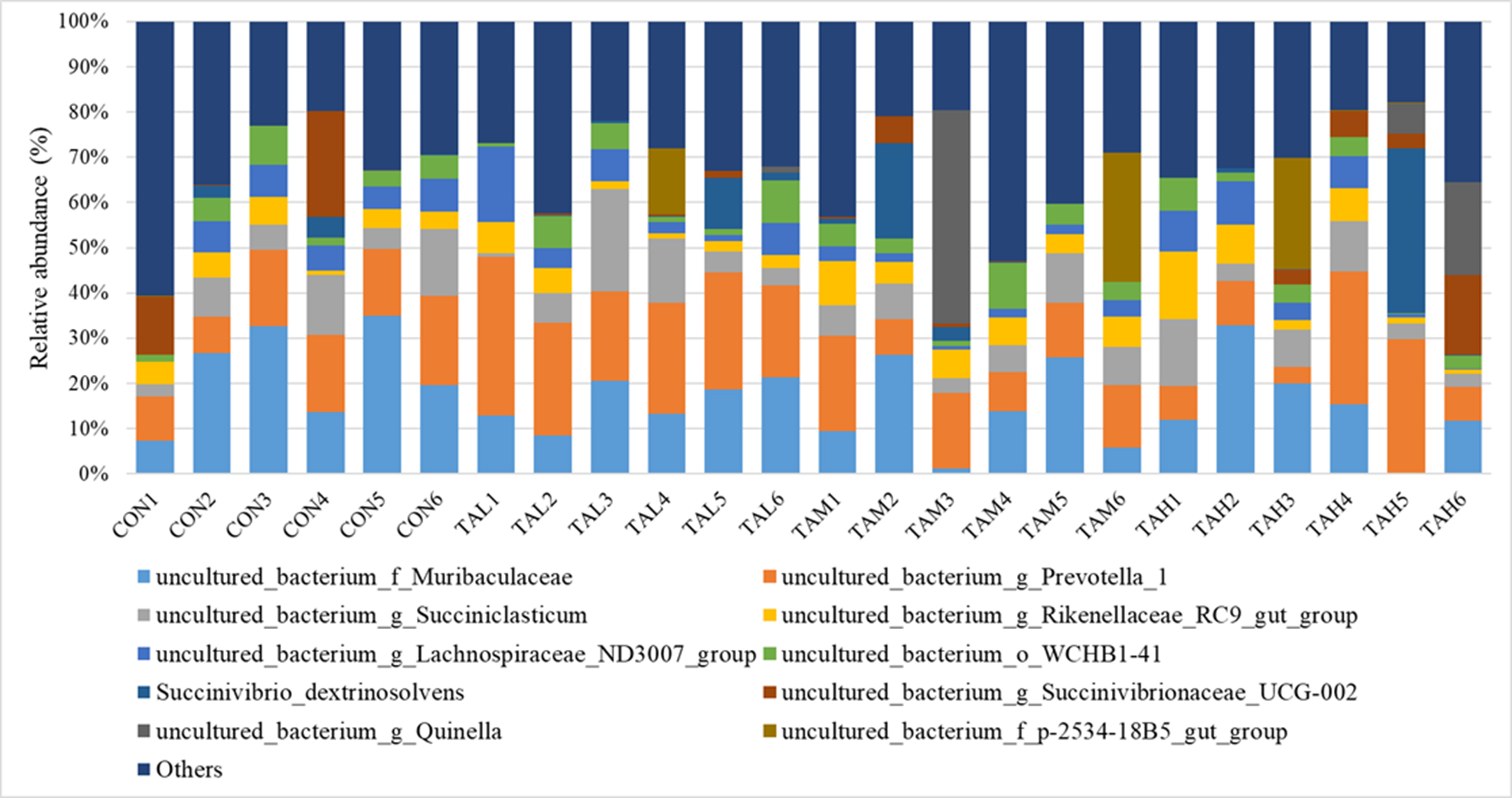

Supplement: Supplementary file 7 [file Image_3.jpg]
